# Prediction of Aggregate Packing with Tubular Macrocapsules in the Inert Structure of Self-Healing Concrete Based on Dewar’s Particle Packing Model

**DOI:** 10.3390/ma17102455

**Published:** 2024-05-19

**Authors:** Harry Hermawan, Alicia Simons, Silke Teirlynck, Giovanni Anglani, Pedro Serna, Jean-Marc Tulliani, Paola Antonaci, Peter Minne, Elke Gruyaert

**Affiliations:** 1 Department of Civil Engineering, Materials and Constructions, Ghent Campus, KU Leuven, Gebroeders De Smetstraat 1, 9000 Ghent, Belgium; harzhermawan@yahoo.com (H.H.); alicia.simons@hotmail.be (A.S.); silketeirlynck@gmail.com (S.T.); peter.minne@kuleuven.be (P.M.); 2 Instituto de Ciencia y Tecnología Del Hormigón (ICITECH), Universitat Politècnica de València, Camino de Vera S/n, 46022 Valencia, Spain; pserna@cst.upv.es; 3 Department of Structural, Geotechnical and Building Engineering (DISEG), Politecnico di Torino, Corso Duca degli Abruzzi 24, 10129 Torino, Italy; giovanni.anglani@polito.it (G.A.); paola.antonaci@polito.it (P.A.); 4 INSTM Research Unit PoliTO-LINCE Laboratory, Department of Applied Science and Technology (DISAT), Politecnico di Torino, Corso Duca degli Abruzzi 24, 10129 Torino, Italy; jeanmarc.tulliani@polito.it

**Keywords:** self-healing concrete, macrocapsules, particle packing model, voids ratio, aggregates

## Abstract

This paper brings a new insight into understanding the influence of macrocapsules in packing systems, which can be useful in designing the inert structure of self-healing concrete. A variety of tubular macrocapsules, in terms of types and sizes, was used to assess the capsules’ effect in the packing, together with various aggregate types and fractions. The voids ratios (*U*) of aggregate mixtures were evaluated experimentally and compared with the prediction via the particle packing model of Dewar. The packing of coarse particles was found to be considerably affected by the presence of macrocapsules, while no capsules’ effect on the packing of fine particles was attained. A higher capsule dosage and capsule aspect ratio led to a higher voids ratio. In the formulation of the inert structure, the packing disturbance due to capsules can be minimised by increasing the content of fine aggregates over coarse aggregates. Dewar’s model showed a good compatibility with experimental results in the absence of capsules. However, the model needed to be upgraded for the introduction of tubular macrocapsules. Accordingly, the effect of macrocapsules was extensively analysed and a ‘*U* model’ for capsules (with some limitations) was finally proposed, offering a high predicting accuracy.

## 1. Introduction

Research on self-healing concrete is steadily growing with the emergence of innovations in terms of newly developed agents [1,2,3], standardisation of tests for self-healing materials [4,5], self-healing modelling [6,7], and commercialisation pathways [8,9]. The application of self-healing materials in concrete matrices is, however, rather limited, while they are mostly applied in paste and mortar matrices [10]. One of the potential causes of this behaviour is that some agents cannot survive in the harsh concrete environment, raising a concern about the longevity of the healing/sealing system. Nevertheless, advanced technologies allow the storage and preservation of the agent inside vessels via encapsulation processes. Numerous vessels have been developed in the past decade, such as capsules [11,12], aggregates [13,14] and polylactic acid particles [15]. Especially for capsules, there are two distinct technologies, namely micro-encapsulation and macro-encapsulation. The difference mainly lies in the final product, where micro-encapsulation generates microcapsules through a series of chemical processes (i.e., in situ polymerisation, emulsification, etc.), enabling the storage of a tiny amount of agent inside a micro-sized capsule, while macro-encapsulation comprises storage of an agent in bigger-sized capsules, usually up to some centimetres as the maximal dimension. Hermawan et al. [10] mentioned that the capsule parameters (i.e., diameter, thickness and length) played a key role in the self-healing scenario as the capsule should be able to break when a crack propagates. Although this is a vital point in designing the optimal parameters of capsules, often research focuses exclusively on it when assessing capsule-based self-healing systems, neglecting other important factors. In fact, before the capsules break due to cracking in the hardened matrix, it is necessary to ensure that the capsules do not introduce negative effects to the concrete (materials). For instance, in a recent publication by the author [16], it was found that a reduction in concrete compressive strength was noticed after the inclusion of randomly distributed macrocapsules, which was hypothesised to be due to the disturbance of the packing and the presence of ‘weak’ spots in the concrete matrix, created by the capsules themselves. The evaluation of the self-healing mechanism and efficiency of concrete/mortar containing macrocapsules has been formerly addressed in our previous works and can be found in [4,16].

In particular, the packing of solid particles in concrete is of great importance as it governs the fresh and hardened properties as well as the economical aspect of concrete as a commercial product. Aggregates, which constitute 60–75% of the total volume of concrete, represent the majority of the material composition. Optimising the concrete mix design is the key to the production of high-performance concrete, with the focus of maximising the particle density (or minimising the voids ratio) [17,18,19]. Meanwhile, there have been fundamental studies on the theoretical modelling and computer simulation of particle packing, such as the Furnas model [20], the Toufar model [21], the modified Toufar model [22], Dewar’s model [23], the Compressible Packing model by De Larrard [24], Schwanda’s model [25], the Linear Packing Density model [26], and the Linear-Mixture Packing model [27].

To date, more developments have been made in advancing the particle packing concept. Wang et al. [28] employed the Horsfield filling theory to produce ultra-high-performance concrete (UHPC) mixtures. The concept emphasised filling the triangular and quadrilateral voids of primary particles with the secondary sphere-shaped particles of diameters 0.414 times and 0.225 times that of the primary particles. Ding et al. [29] developed a novel ‘divide and fill’ method for generation of particle packing via numerical simulation. The method relied on controlling the particle shape, distribution and grading to achieve the optimal particle filling. Yanzhou et al. [30] used the Dinger–Funk particle size distribution (PSD) equation, derived from the modified Andreasen model, to achieve a densely compacted concrete mixture. Sobolev et al. [31] successfully developed the Sequential Packing Algorithm (SPA) to model the real particulate systems used in concrete composed of one million particles that differ in size by up to 150 times. Dingqiang et al. [32] compared the packing approaches via a statistical model based on D-Optimal Mixture Design (DOMD) and a Genetic Algorithm-based Artificial Neural Network (GA-ANN) for designing UHPC. It was found that the GA-ANN exhibited a higher accuracy and a higher practicability than DOMD.

Moreover, there have been efforts in understanding the packing of particles mixed with fibres for fibre-reinforced concrete (FRC) applications [33,34,35]. The characteristics of the fibres, either rigid or flexible, will greatly affect the packing, as the rigid fibres cannot deform to fill the voids while the adverse effect may be obtained using flexible fibres [36]. In general, the rigid fibres cause a wall effect in the packing, inducing more voids between particles and fibres than between the particles themselves. Despite the physical differences, the macrocapsules may be considered as a kind of large-sized rigid fibre for the packing study. In fact, the packing of aggregate–capsule mixtures is more complicated than that of aggregate mixtures as the capsules may also interfere with the packing of granular materials and they cannot be treated as equivalent to aggregates. Aside from the aforementioned drawback on mechanical performance, the addition of macrocapsules also causes a reduction in workability (reduced slump and increased air content) [16] and the authors linked this tendency partially to an adverse effect of the capsules on the aggregate packing. Nevertheless, at the time this paper was written, no single study had investigated the effect of capsules on the packing, which strongly motivated the authors to fill this research gap.

The research presented herein is the first phase in understanding the effect of macrocapsules in the aggregate packing, which can be useful for designing capsule-based concrete mixtures. Several types and sizes of macrocapsules and aggregates were used to determine the voids ratios of capsule–aggregate mixtures. The particle packing model of Dewar was employed to construct interaction diagrams between the voids ratio and fine fraction of aggregate mixtures with and without capsules. The main objectives of this study were (i) to assess the alteration in aggregate packing due to the introduction of macrocapsules and (ii) to adapt Dewar’s model with capsules’ parameters to predict the voids ratio of aggregate–capsule mixtures. It is noteworthy that the work conducted in this area is new and a comparison with the literature was not possible at the time this paper was written. Further, this paper does not consider the self-healing mechanism and the interface strength between the tubular capsule and cementitious matrix as the current research is still in the design phase.

## 2. Particle Packing Model of Dewar

The particle packing method based on the voids ratio of materials was introduced by J.D. Dewar [23] in 1999. The voids ratio is generally defined as the ratio of total void volume to total solid volume, and it is a function of the shape and surface texture of particles and of the grading in relation to the mean size of particles. In the case of a two-size particle mixture, the mixing of fine and coarse particles dilates the structure of the coarse particles. It assumes that the coarse particles move apart to occupy the centres of spaces and the coarse particles are spaced apart *m* times the mean size of fine particles (*D*_1_). The effective voids ratio of the coarse particles ($U_{0}^{\prime \prime }$) when the structure is dilated is calculated as [23]:

(1) $$U_{0}^{\prime \prime }=\left(1+U_{0}\right){\left(1+mr\right)}^{3}-1$$

where $U_{0}$ = voids ratio of coarse particles, m = spacing factor, r = ratio of mean sizes = $D_{1}$/$D_{0}$ and $D_{0}$ is the mean size of coarse particles.

**Figure 1:**
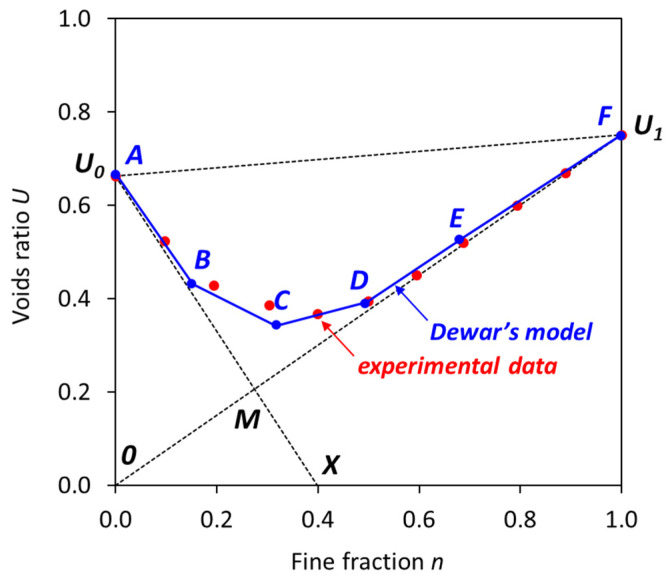
Theoretical voids ratio diagram.

A typical voids ratio or interaction diagram of two materials is illustrated in Figure 1, where the x-axis represents the fine fraction (*n*) of materials on a volumetric basis and the y-axis is the voids ratio (*U*). The voids ratio of all coarse particles (*U*_0_) is located at the left vertical axis when *n* equals 0, while the voids ratio of all fine particles (*U*_1_) is located at the right vertical axis when *n* equals 1. The line *U*_1_0 represents the theoretical effect of the addition of coarse material to fine material without increasing the voids ratio of the fine material. The line *U*_0_*X* represents the theoretical effect on the voids ratio of adding fine material without any dilation of the structure of the coarse material. *M* is the point at which the voids in the coarse material are completely filled with the fine material at its own voids ratio while the coarse particles remain in contact. The triangular area 0*MX* represents hypothetical mixtures which cannot exist. The triangular area *U*_0_*MU*_1_ represents all practical mixtures (see Figure 1, experimental data) that can exist in the presence of particle interference. The lower boundary *U*_0_*MU*_1_ represents the voids ratios for all combinations of the two materials that could exist in the absence of particle interference. The upper boundary *U*_0_*U*_1_ represents the case when the mean sizes of the materials are equal, and the particle interference is at its maximum. Powers [37] described the particle interference as the disturbance of the structure of the finer particles by the larger particles, which causes an increase in the voids content. De Larrard [24] further elaborated that the ‘aggregate wall’ effect and ‘loosening’ effect are responsible for the particle interference. The aggregate wall effect is defined as a phenomenon when an isolated coarse particle disturbs the packing and increases the voids surrounding this coarse particle, while the loosening effect is a phenomenon when an isolated fine particle in the structure of coarse particles appears to be too large to fit in the space between coarse particles, thus disturbing the packing.

According to the particle packing model of Dewar, the voids ratio diagram of the combined materials as a function of the fine fraction can be constructed using several change points (denoted as A to F) as presented in Figure 1. All change points are joined by straight lines. These change points are associated with the material properties of certain fractions, the spacing factor *m*, and empirical factors *k_int_* and *k_p_* from Table 1. The voids ratio (*U_n_*) and fine fraction (*n*) of each change point can be calculated by the following equations [23]:

(2) $$U_{n}=nU_{1}^{\prime \prime }$$

(3) $$U_{1}^{\prime \prime }=\frac{(1+U_{1})U_{0}^{\prime \prime }}{\left(1+U_{0}^{\prime \prime }\right)-{\left(1+Z\right)}^{3}}-1$$

(4) $$n=\frac{U_{0}^{\prime \prime }}{1+U_{1}^{\prime \prime }+U_{0}^{\prime \prime }}$$

(5) $$Z=k_{int}+\left[{\left(1+U_{0}\right)}^{1/3}-1-k_{int}\right]r^{k_{p}}$$

where $U_{1}^{\prime \prime }$ is the effective voids ratio of fine particles, $U_{0}^{\prime \prime }$ is the effective voids ratio of coarse particles, r = the ratio of mean sizes and Z is the notional width factor. In Figure 1, the line A–B represents a partial filling of voids of coarse particles by fine particles and the particle interference is minimal. The major interference in the packing of both fine and coarse particles occurs in the lines B–C and C–D. The lines D–E and E–F represent minor interference within fine and coarse particles.

## 3. Materials and Methods

### 3.1. Tubular Macrocapsules

To determine the effect of capsule parameters in the packing of aggregates, two types of tubular macrocapsules were used, namely (1) cementitious capsules and (2) polymeric capsules.

**Figure 2:**
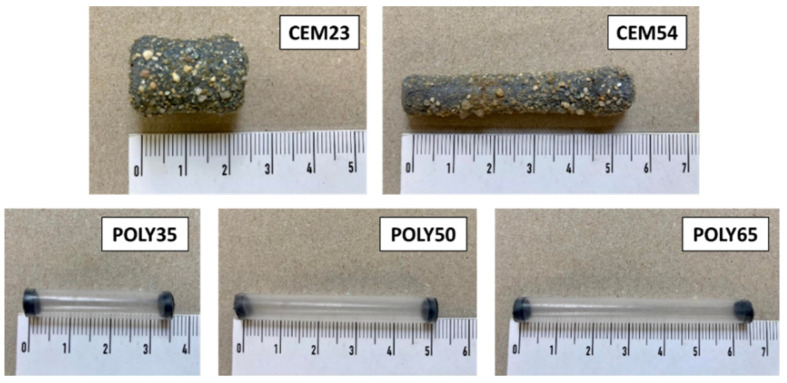
Types of macrocapsule used in this study.

The cementitious capsules were mainly made of cement and chemical compounds, and were externally coated with an epoxy and a sand layer for the shock resistance of the shell and the conservation of the cargo [38]. The detailed composition and the manufacturing process of these capsules can be found in [4]. Short cementitious capsules and long cementitious capsules were used with an average length of 23 and 54 mm, respectively (hereinafter referred to as CEM23 and CEM54). The physical appearance of both capsule types is shown in Figure 2 and the average geometric properties of the capsules are summarised in Table 2. As a note, the volume listed in Table 2 considers the full volume of the capsule based on the mean length and the mean outer diameter.

The polymeric capsules were prepared from FEP (Fluorinated Ethylene Propylene)-extruded tubes supplied by ZEUS. These tubes were chosen due to their good rigidness and because they did not deflect when they were combined with aggregates. The tubes were cut into three different lengths of 35, 50 and 65 mm (hereinafter referred to as POLY35, POLY50 and POLY65) and all capsules were closed with rubber at both ends (see Figure 2). The average geometric properties of these capsules are also reported in Table 2. It should be noted that the polymeric capsules are not intended to be used for self-healing concrete applications because in some cases there could be incompatibility issues between the polymeric capsule and the stored healing agent, causing premature hardening or polymerisation of the healing agent inside the capsule [39]. The polymeric capsules were mainly used as a proof-of-concept to further validate the influence of capsule parameters on the packing of aggregates, in addition to the cementitious capsules (which have already been demonstrated to be compatible with most healing agents and with the concrete matrix [4,16,38]).

### 3.2. Aggregates

**Figure 3:**
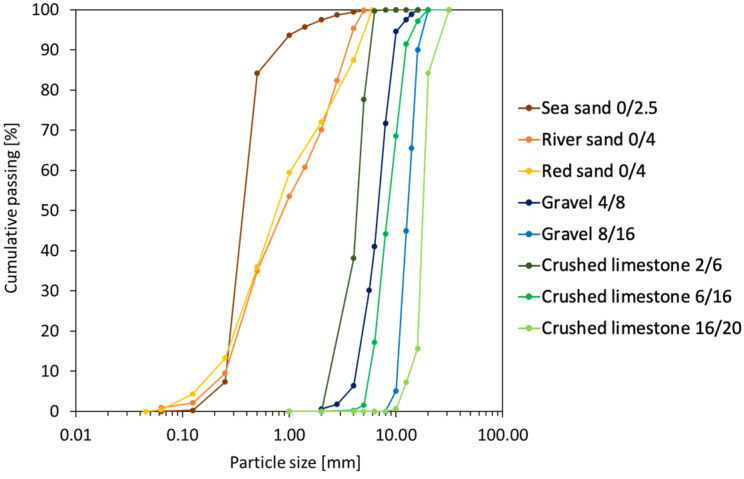
Particle size distribution of aggregates.

Several types of aggregate were employed in this study, starting from the fine to the coarse fractions. The main objective was to combine aggregates and capsules at different dosages in order to analyse the effect of capsules in the packing of a certain aggregate type/fraction. The properties of aggregates can be found in Table 3 as determined by particle density tests (EN 1097-6 [40]) and loose bulk density tests (EN 1097-3 [41]), and the particle size distributions (PSD) of all aggregates are depicted in Figure 3 as determined by sieving tests (EN 933-1 [42]).

### 3.3. Testing Method

The loose bulk density (LBD) test was mainly performed in this study in accordance with EN 1097-3 [41]. The test was conducted by filling the container with dried aggregates until it was fully filled without any compaction. Via this test, the loose bulk density ($\rho_{b}$) can be measured and the parameter of interest, the voids ratio (*U*), can be determined as follows [23]:

(6) $$\;\;\;\;\;\rho_{b}=\frac{m_{2}-m_{1}}{V}\times 10^{3}$$

(7) $$U=\frac{\rho_{rd}}{\rho_{b}}-1$$

where $\rho_{rd}$ is the oven-dried density of aggregates [kg/m^3^], $m_{2}$ is the mass of the container and oven-dried material [kg], $m_{1}$ is the mass of the empty container [kg] and *V* is the volume of the container [dm^3^]. In this study, a steel container of 7.5 L was employed for all types of aggregates. The parameter of *U* is a great of importance as it elucidates the packing system by considering the ratio of total void volume to total solid volume.

Furthermore, the capsules were combined with the aggregates at specific dosages ranging from 0 to 3.5% *v*/*v* (see Table 4). The unit of % *v*/*v* is defined as the volume of capsules over the volume of the container (as a note, the volume of the container represents the volume of aggregates + volume of voids + volume of capsules). For CEM23 and CEM54 capsules, the capsule dosage was fixed at approximately 0, 0.5, 1.0, 2.1 and 3.2% *v*/*v*, which means that a different number of capsules was assigned. The same was applied for the addition of POLY capsules, where the dosage was fixed between 0 and 3.5% *v*/*v*. Although the number of capsules was varied depending on the size/volume of the corresponding capsules, the capsule dosage was relatively comparable both for CEM and POLY capsules. It shall be noted that this study considers randomly distributed capsules amongst aggregate particles. The effect of capsules in the packing system can be identified directly on the changes in the voids ratio of aggregates. Therefore, the loose bulk density of aggregates due to the presence of capsules can be calculated as follows:

(8) $$\rho_{b}=\frac{m_{3}-m_{1}-m_{caps}}{V-V_{caps}}\times 10^{3}$$

where $m_{3}$ is the mass of the container, oven-dried test specimen and capsules [kg], $m_{caps}$ is the mass of added capsules [kg] and $V_{caps}$ is the volume of added capsules [dm^3^].

**Figure 4:**
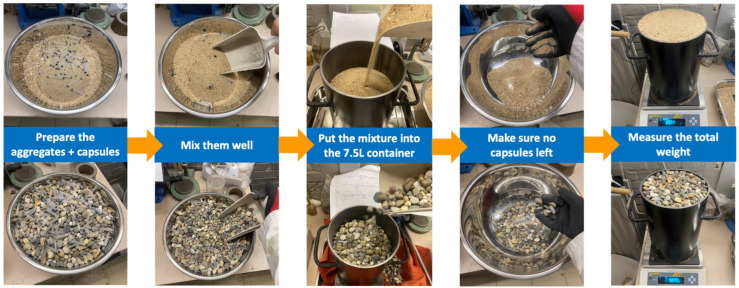
Schematic procedure of LBD test on a single type/fraction of aggregate.

**Figure 5:**
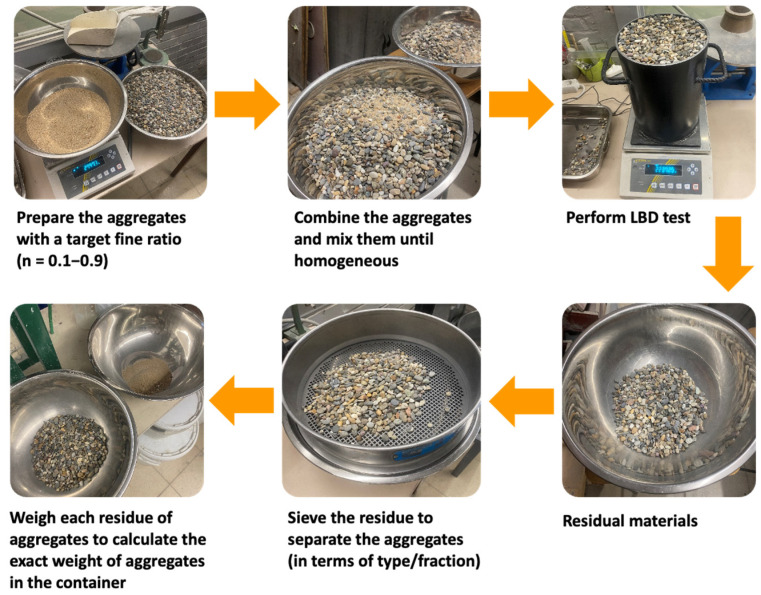
Schematic procedure of the LBD test on aggregate mixtures.

A schematic procedure of the LBD test on a specific aggregate type/fraction can be found in Figure 4. The procedure for the use of a mixture of aggregates (e.g., sand and gravel) with a specific fine fraction (*n*) is presented in Figure 5. The fine fraction was tested from 0.1 to 0.9, which was based on the volumetric ratio. The voids ratio of mixed fine and coarse aggregates can be calculated by the theoretical oven-dried density of the mixture $\rho_{rd\_n}$ (see Equation (9), with the oven-dried density of the fine fraction $\rho_{rd\_1}$ and coarse fraction $\rho_{rd\_0}$ [41]) and the experimental loose bulk density of the mixture.

(9) $$\rho_{rd\_n}=n\rho_{rd\_1}+(1-n)\rho_{rd\_0}$$

As a note, three repetitions were used for each LBD test and the mean value of the measurements was denoted the experimental voids ratio. Moreover, these aggregate mixtures were mainly tested as follows:

- Binary aggregate mixture (BAM): gravel 4/8 + gravel 8/16;
- Ternary aggregate mixture (TAM): sea sand 0/2.5 + (gravel 4/8 + gravel 8/16 with *n* = 0.65).

## 4. Results and Discussion

### 4.1. Voids Ratio of Aggregates

The voids ratio of a single aggregate type/fraction without the addition of macrocapsules was initially measured and the result is tabulated in Table 3. It is clear that the voids ratios were different for all aggregates and mainly depend on the fraction or particle size distribution of the respective aggregates, and the shape and roughness of particles. These initial *U* values were regarded as the basis for the evaluation of the changes in *U* as a function of capsule dosage. Specifically, here, only cementitious capsules (CEM23 and CEM54) were used for the initial assessment. The CEM capsules were added to different types of aggregates (i.e., sea sand, river sand, red sand, gravel and crushed limestone) and different fractions of aggregates (i.e., 0/2.5, 0/4, 2/6, 4/8, 6/16, 8/16 and 16/20).

**Figure 6:**
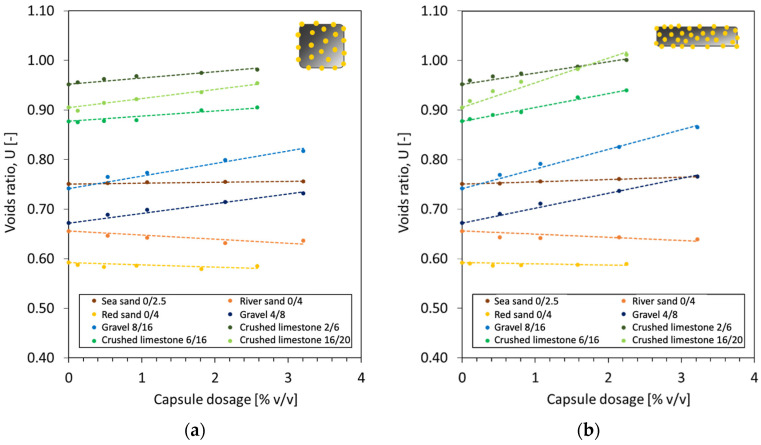
Voids ratio of different types and fractions of aggregates as a function of the capsule dosage of (**a**) CEM23 and (**b**) CEM54.

**Figure 7:**
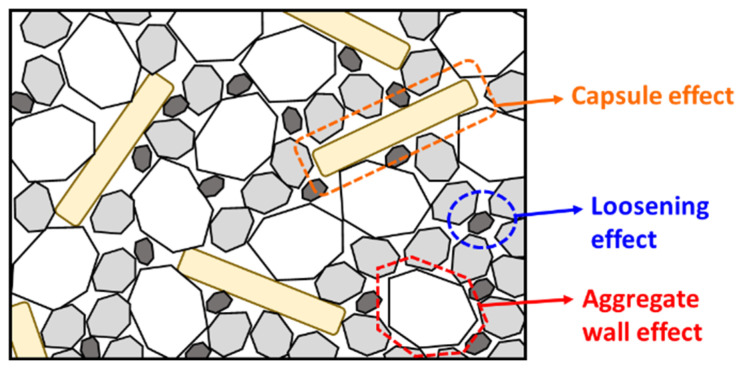
Capsule effect in the packing of aggregates.

The development of *U* as a function of capsule dosage is summarised in Figure 6 with implementation of a linear regression. It is obvious that the voids ratio of fine aggregates (i.e., sea sand, river sand, red sand) did not truly change after the addition of CEM capsules at any dosage (either the case of CEM23 or CEM54), which is shown by a flat linear regression line (nearly zero slope). Contrarily, the voids ratio of coarse aggregates (i.e., gravel and crushed limestone) considerably increased as the capsule dosage increased. The increase in voids content can be explained due to the secondary loosening and wall effects induced by the capsules, referred to as the capsule effect, as illustrated in Figure 7. This capsule effect generates additional voids surrounding the perimeter of the capsule wall/shell when in contact with aggregates and loosens the packing between each particle.

In order to assess the effect of different CEM capsules, a factor *k* was introduced as the slope of the percentage change in the voids ratio versus the dosage of capsules, and the result is tabulated in Table 5. Apparently, the *k* values with CEM23 were always lower than the *k* values with CEM54, meaning that the voids ratio of aggregates was more affected by long capsules than short capsules. This is logical because the shape of short capsules is comparable to that of the round aggregates, so they can be blended better with aggregates. On the other hand, the long capsules are available in a rod-like shape. Thus, when these capsules are mixed with aggregates, the capsules induce extra voids in the packing due to the increased gaps between the long capsules and aggregates as compared with the use of short capsules.

The ratio of *L_caps_*/*D_caps_* may also play a role in the changes in *U*. The increase in *L_caps_*/*D_caps_* with a factor 3.9 (from 1.52 (CEM23) to 5.98 (CEM54)) caused an increase of *k* by 54–56% on all types of gravel and 84–176% on all types of crushed limestone. The effect of capsules was also higher with the coarser fractions of aggregates than with the smaller fractions. Based on Table 5, the increment of *k* was higher for the use of crushed limestones than the use of gravels, showing that the effect of capsules is greater when combined with crushed limestones. The physical characteristics of coarse aggregates also influence the voids ratio of aggregates in the interaction with the capsules. Gravel is typically characterised by its rounded shape and smooth texture, while crushed limestone is characteristically jagged, with sharp and pointy edges and a rough surface. Gruyaert et al. [39] previously reported that crushed limestone exerted a higher impact on the (polymeric) capsules than gravel in terms of the capsules’ resistance during concrete mixing. By taking into account these observations, it was decided to further analyse the effect of capsules in the packing of specific aggregates, including sea sand 0/2.5, gravel 4/8 and gravel 8/16.

**Figure 8:**
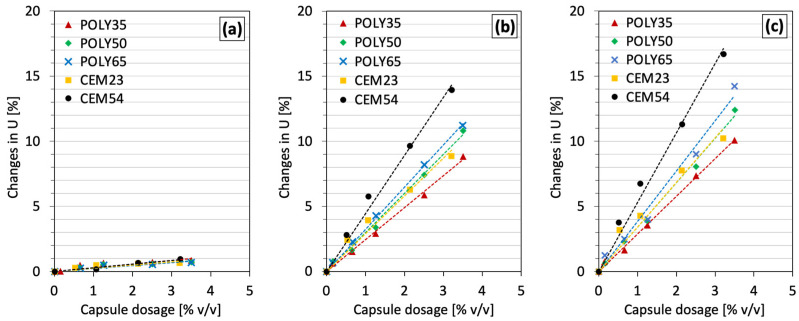
Changes in voids ratio due to the increase in capsule dosage with a certain aggregate: (**a**) sea sand 0/2.5, (**b**) gravel 4/8 and (**c**) gravel 8/16.

The effect of POLY capsules was also investigated, and the results are presented in Figure 8. Similarly to CEM capsules, a scant effect of POLY capsules was observed on the voids ratio of fine aggregates (see Figure 8a). The *k* values for all capsules with sand 0/2.5 were nearly identical, around 0.2, confirming no substantial disturbance of the packing of fine aggregates. According to Figure 8b,c, increasing the amount of POLY capsules gradually increases the voids ratio of coarse aggregates, for instance, the voids ratio of gravel 4/8 increased by 1.7%, 3.4% and 7.4% with the use of POLY50 at 0.6% *v*/*v*, 1.2% *v*/*v* and 2.5% *v*/*v*, respectively. With the use of a coarser fraction such as gravel 8/16, the presence of capsules exhibited higher *k* values than for gravel 4/8. On the other hand, there was a gradual increase in *k* as the length of POLY capsules increased from 35 to 65 mm, which was observed at all dosages.

**Figure 9:**
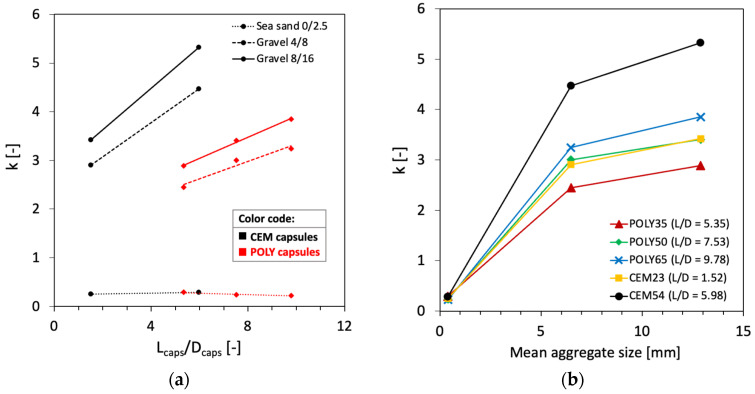
(**a**) Relationship between *k* and *L_caps_*/*D_caps_* observed with the use of specific capsule types (note: black line/dot is based on CEM capsules, while red line/dot is based on POLY capsules); (**b**) relationship between *k* and mean aggregate size observed with the use of specific capsule types.

A relationship between the *L_caps_*/*D_caps_* ratio and the *k* factor was established, as shown in Figure 9a. It is clear that there is a linear relationship between *L_caps_*/*D_caps_* and *k*, as observed from the results based on POLY capsules. The *k* values remained constant on sand particles with different *L_caps_*/*D_caps_*, while the *k* increased linearly as *L_caps_*/*D_caps_* of coarse particles increased. Although there were only two points for CEM capsules (see Figure 9a in black), the results can be assumed to be linear by taking into account the findings from the POLY capsules (Figure 9a, red).

Moreover, Figure 9b shows that the mean size of aggregates influences the *k* values, where the bigger the mean aggregate size, the higher the *k* values. The aforementioned results generate information about the relationship of three potential parameters, namely *k*, mean aggregate size (*D_agg_*) and *L_caps_*/*D_caps_*, for modelling the effect of capsules in the packing of aggregate. The selection of aggregate and the *L_caps_*/*D_caps_* ratio of CEM capsules will play key roles in controlling the packing density.

**Figure 10:**
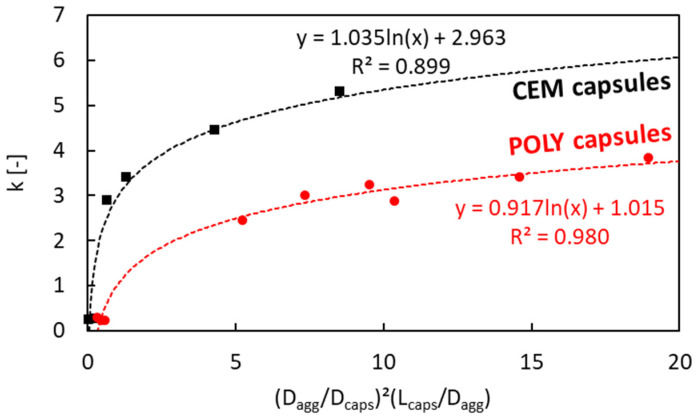
Factor *k* versus (*D_agg_*/*D_caps_*)^2^(*L_caps_*/*D_agg_*) (only considering the following aggregates: sea sand 0/2.5, gravel 4/8 and gravel 8/16).

As there is no available model to determine the macrocapsules’ effect, the macrocapsules used in this study were seen and modelled as steel fibres. Chu et al. [36] previously developed a packing model of rigid fibres on aggregate packing using a relationship between factor *k* and (*G*/*D_fibre_*)^2^(*L_fibre_*/*G*)*^α^* to set a limit on the aggregate size to avoid an excessive increase in the voids ratio when steel fibres are added. *G* is defined as the geometric mean size of the aggregate. The *G* value equals the mean size of the aggregates (*D_agg_*) as defined by Dewar. The power of 2 was obtained from the study of Chu et al. considering the presence of particles in close proximity to the fibres, while the *α* coefficient was obtained at 1.5 after performing a linear regression with an *R*^2^ of 0.96. The same modelling procedure was followed with the use of the current results based on the macrocapsules and the relationship between *k* and (*G*/*D_fibre_*)^2^(*L_fibre_*/*G*)*^α^*. It was found that, in our case, the best fitting of the *α* coefficient was found at 1.0 (*R*^2^ = 0.89). However, the linear regression model of steel fibres cannot be implemented in the model of macrocapsules and it seems that a logarithmic regression fits best with our results as shown in Figure 10. This may be due to the different physical interactions between the steel fibres and macrocapsules with the aggregate particles. In Figure 10, two logarithmic regressions are presented: one for CEM capsules and the other for POLY capsules. Originally, the aim was to generalise the results based on the change in *L_caps_*/*D_caps_* for either POLY or CEM capsules (refer to Figure 9a). However, the regression result seemed to not be reliable, and it was hypothesised that there is a completely different interference level between those capsules and the aggregates.

### 4.2. Interaction Diagram of Binary Aggregate Mixture (BAM)

The initial results showed that the presence of capsules did not disturb the packing of fine aggregates and it is more important to assess the effect of capsules in the packing of combined coarse aggregates. Therefore, the study of the interaction of an aggregate mixture was started from a mixture of coarse particles (here gravel 4/8 + gravel 8/16), further called the binary aggregate mixture (BAM). In the next stage, a ternary aggregate mixture (TAM) was made, employing a combination of (sea sand 0/2.5 + (gravel 4/8 + gravel 8/16)).

**Figure 11:**
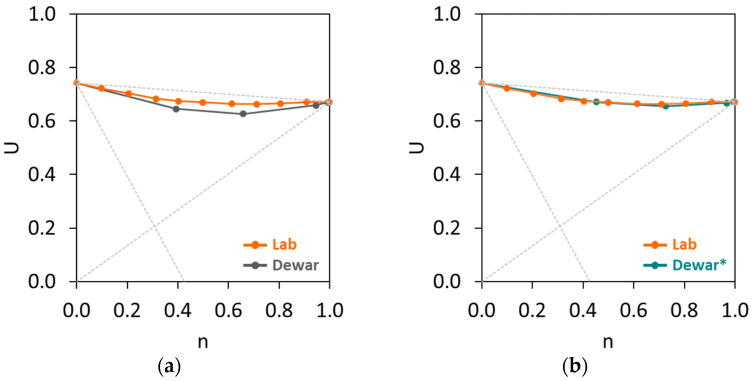
Voids ratio of BAM (without capsules): (**a**) original result, (**b**) Dewar result with adjustment factor *F* = 1.3.

The BAM was initially made by combining two fractions of gravel, and the fine fraction was tested from 0.1 (90% gravel 8/16 + 10% gravel 4/8) to 0.9 (10% gravel 8/16 + 90% gravel 4/8). The loose bulk density test results allowed the calculation of the voids ratios, as presented in Figure 11, comparing the lab result and Dewar’s model. As shown in Figure 11a, the differences between the *U* values of BAM at all fractions were relatively small, where the *U* ranged from 0.742 (*n* = 0) to 0.672 (*n* = 1). The lowest *U* was found at a fine fraction between 0.6 and 0.7. Theoretically, the interaction diagram of BAM closely reaches the straight line of *U*_0_*U*_1_, which means that there is a high particle interference in this aggregate mixture. Dewar’s model was employed in this BAM in order to analyse the conformity between the experimental (lab) result and Dewar’s result. Figure 11a evidently shows that there is a slight mismatch between lab and Dewar results. The packing theory of Dewar assumes that a graded material can be represented by a single-sized material having the same voids ratio and mean size. In case of combining materials with different fractions, the material shall be arranged from the finest to the coarsest particle. The implementation of single-sized fractions to graded fractions could result in the inaccurate estimation of the fraction mean size and further affect the size ratio *r* (ratio of the smaller mean size to the bigger mean size of the fractions of an aggregate mixture) [43]. In addition, the accuracy of Dewar’s model mainly relies on the basic inputs used in the model. The estimation of mean aggregate size based on the particle size distribution from the sieving test can also be inaccurate. Therefore, Dewar [23] proposed an adjustment factor *F* to improve and calibrate the predicting accuracy of the model on the size ratio *r* (resulting in *F* × *r*). Based on trial and error in different single-sized and multi-sized aggregate mixtures, Dewar [23] set the limit of *F* between 0.6 and 1.6. In this study, Dewar’s model in BAM was calibrated with an *F* of 1.3 and the result can be found in Figure 11b. The adjustment factor clearly helps to improve the model’s fit with the lab result, with a good accuracy. Depending on the used mixtures, a different adjustment factor may be necessary to avoid discrepancies between the experimental voids ratios and the predictions from Dewar’s model, as previously discussed by Liu et al. [43].

**Figure 12:**
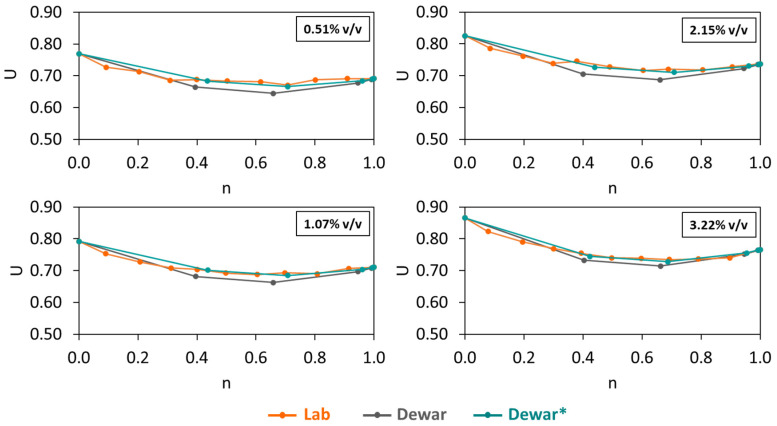
Voids ratios of BAM with CEM54 capsules at different dosages (note: ‘Lab’ represents experimental results (orange line), ‘Dewar’ is Dewar’s result without adjustment factor (grey line), ‘Dewar*’ is Dewar’s result with adjustment factor *F* = 1.1–1.2 (green line)).

Moreover, BAM was initially combined with CEM54 capsules at different dosages at 0.51, 1.07, 2.15 and 3.22% *v*/*v* and the *U* results are shown in Figure 12. Similar to the case of 0% CEM54 capsules, there were minor differences between the experimental results and Dewar results. To increase the prediction accuracy of Dewar’s model, the adjustment factor was also applied to all mixtures with CEM54 capsules with an *F* of 1.1–1.2 (which was similar to the *F* of BAM without capsules). After implementing *F*, *r* of each BAM mixture with capsules was corrected and the result of the adjusted Dewar (Dewar*) model showed a good fitting capability, with results close to the experimental result. Comparing Figure 11 and Figure 12, the interaction diagrams of BAM without and with capsules displayed a similar trend.

**Figure 13:**
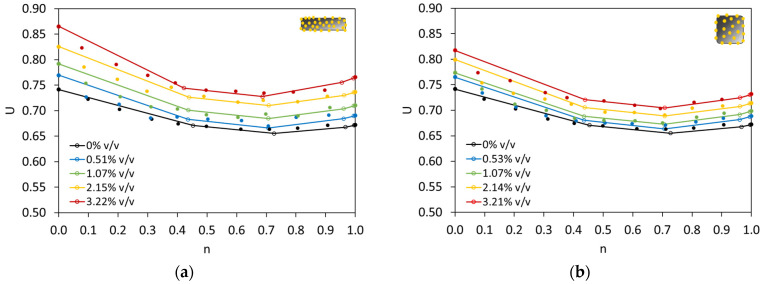
Voids ratios of BAM with (**a**) CEM54 capsules and (**b**) CEM23 capsules at different fine fractions (note: the straight line with hollow dots represents the Dewar* result and solid dots represent the experimental/lab result).

To showcase the capsules’ effect, the above results are summarised in Figure 13a. It is obvious that the addition of CEM54 capsules increased the voids ratio of BAM at any fraction. A large increase in *U* due to capsules was mainly observed at *n* = 0, and when *n* was in the range of 0.4–0.7, the capsules’ effect was lower than that at *n* = 0. This can be explained because, within the aforementioned range of *n*, BAM is well packed with the lowest *U* values among others, and a sufficient quantity of fine particles is available to fill the voids. Based on the experimental result, the lowest *U* occurred at *n* of 0.6 or 0.7. Next, Dewar’s model was implemented with the aid of an adjustment factor, and the results showed that not all experimental results really fit with the prediction of Dewar’s model, especially for *n* < 0.4. However, for *n* ≥ 0.4, Dewar’s results were relatively reliable. Following the current objective of finding the optimal packing of BAM, Dewar’s model indicated *n* of 0.7 as the optimal fine fraction, having the lowest *U*. It shall be noted that Dewar uses a few points (i.e., A–F; 6 predicted points) in the interaction diagram to model the packing, while for experimental tests, *n* was tested from 0 to 1 (11 actual points). Nevertheless, the lowest *U* predicted by Dewar’s model (0.7) was similar to the experimental result (0.6–0.7), showing a good accuracy of Dewar’s model to predict the optimal packing with the aid of *F*. The same analysis was also performed on the use of short capsules (CEM23), which is shown in Figure 13b. Comparing the results from CEM54 and CEM23 capsules (see Figure 13a vs. Figure 13b), the gap between the interaction diagrams at all capsule dosages was less pronounced on CEM23 than on CEM54. This reflects that short capsules may induce less disturbance in the packing of BAM than long ones.

**Figure 14:**
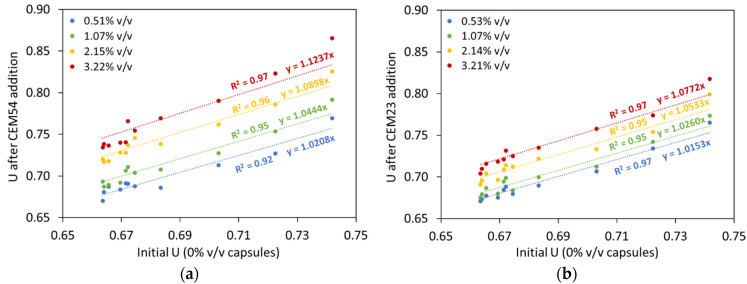
Changes in the voids ratio of BAM after the addition of (**a**) CEM54 capsules and (**b**) CEM23 capsules compared to the initial voids ratio of BAM without capsules.

**Figure 15:**
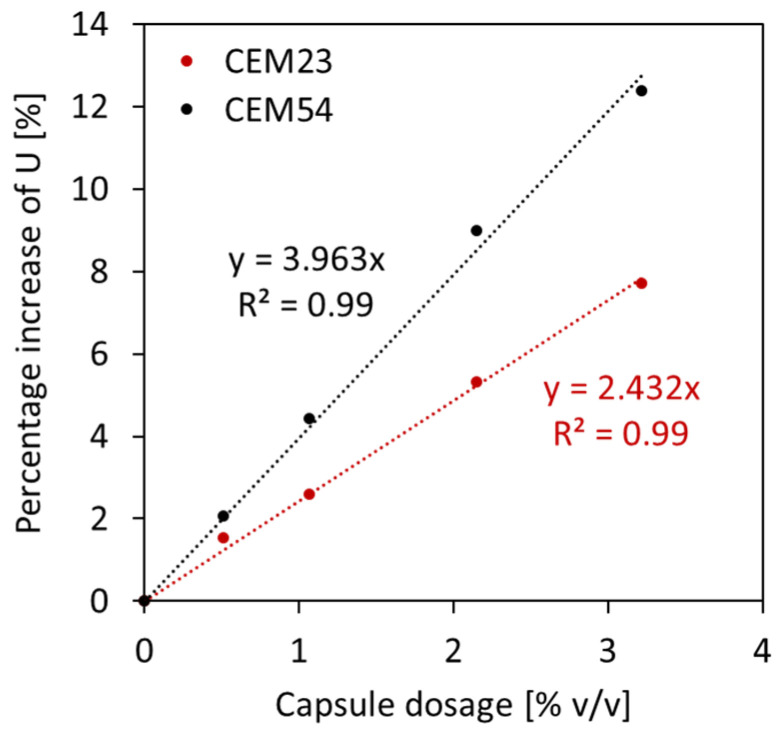
Relationship between capsule dosage and percentage increase in voids ratio observed in BAM.

**Figure 16:**
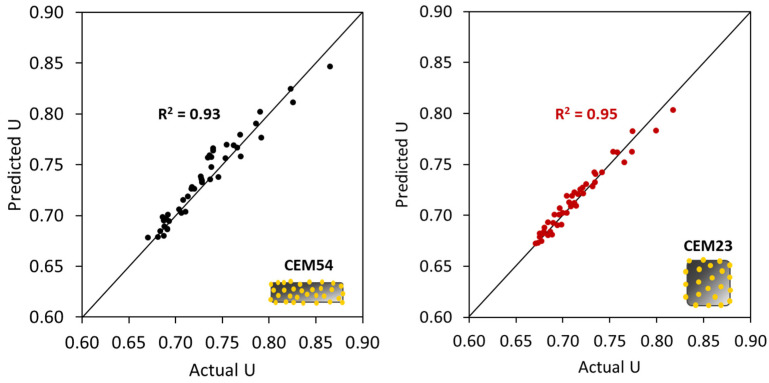
Comparison between the actual *U* values and the predicted *U* values of BAM as a function of capsule dosage and *L_caps_*/*D_caps_* ratio.

In order to further understand the results from Figure 13, a relationship between the voids ratio at 0% *v*/*v* capsules and the voids ratio at a certain capsule dosage was constructed (see Figure 14). It is evident that the voids ratio of BAM increased with increasing the capsule dosage. The percentage increment in *U* toward capsule dosage could be established, as depicted in Figure 15. It was confirmed that the effect of long capsules was more dominant than that of short capsules. Based on these extensive analyses, the following formula is proposed to predict the voids ratio of BAM for the addition of capsules:

(10) $$U_{BAM\_caps}=U_{BAM\_initial}\left((0.004\frac{L_{caps}}{D_{caps}}+0.02)d_{caps}+1\right)$$

where $U_{BAM\_caps}$ is the voids ratio of BAM after capsules’ addition, $U_{BAM\_initial}$ is the initial voids ratio of BAM without any capsules, $d_{caps}$ is the dosage of capsules [% *v*/*v*], $L_{caps}$ is the length of the capsule [mm], and $D_{caps}$ is the outer diameter of the capsule [mm]. Figure 16 shows that the above equation can be used to predict the voids ratio of BAM as a function of the capsule dosage and *L_caps_*/*D_caps_* ratio with good accuracy.

### 4.3. Interaction Diagram of Ternary Aggregate Mixture (TAM)

**Figure 17:**
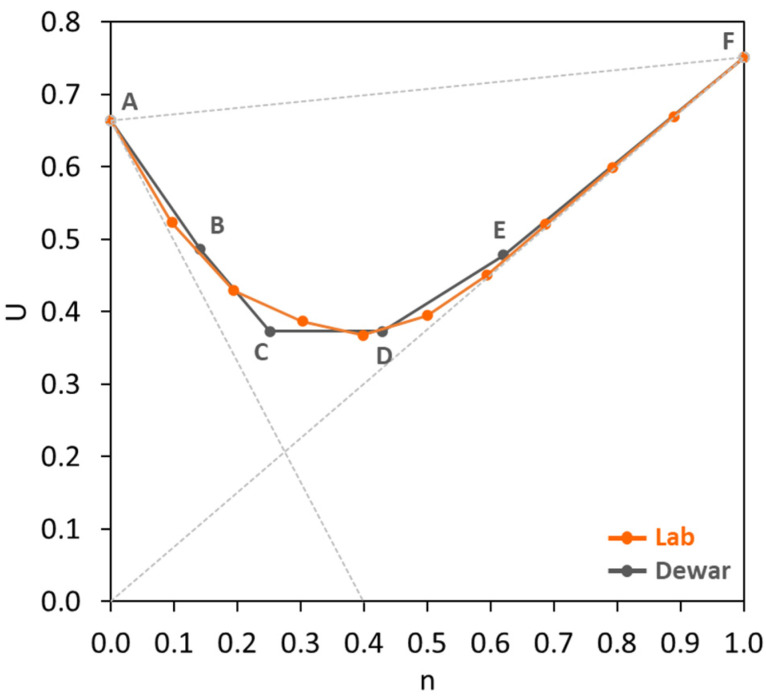
Voids ratio of TAM (without capsules) (note: Lab is experimental result (orange line) and Dewar is Dewar’s result without adjustment factor (grey line)).

TAM was made by employing a combination of (gravel 4/8 + gravel 8/16) and sea sand 0/2.5. A mixture of gravels was selected based on the analysis of BAM. As previously discussed in the interaction diagram of BAM, the lowest voids ratio was found with *n* in the range of 0.6–0.7 based on the experimental result, and at *n* of 0.7 based on Dewar’s result. It was decided to take the *n* of 0.65 as an average fine ratio, meaning that the coarse mixture consisted of 65% gravel 4/8 and 35% gravel 8/16 in vol%. The coarse mixture and sand were gradually mixed starting from *n* of 0.1 (90% (gravel 4/8 + gravel 8/16 with *n* = 0.65) + 10% sea sand 0/2.5) to 0.9 (10% (gravel 4/8 + gravel 8/16 with *n* = 0.65) + 90% sea sand 0/2.5). The LBD tests were performed, and the interaction diagram of TAM is presented in Figure 17. It is obvious that the lab result matches very well with the theoretical model of Dewar, even without the aid of an adjustment factor. It should be noted that not all mixtures require an adjustment factor, and this depends mainly on the sensitivity of the LBD test and the narrow/broad particle size distribution of the used aggregates.

**Figure 18:**
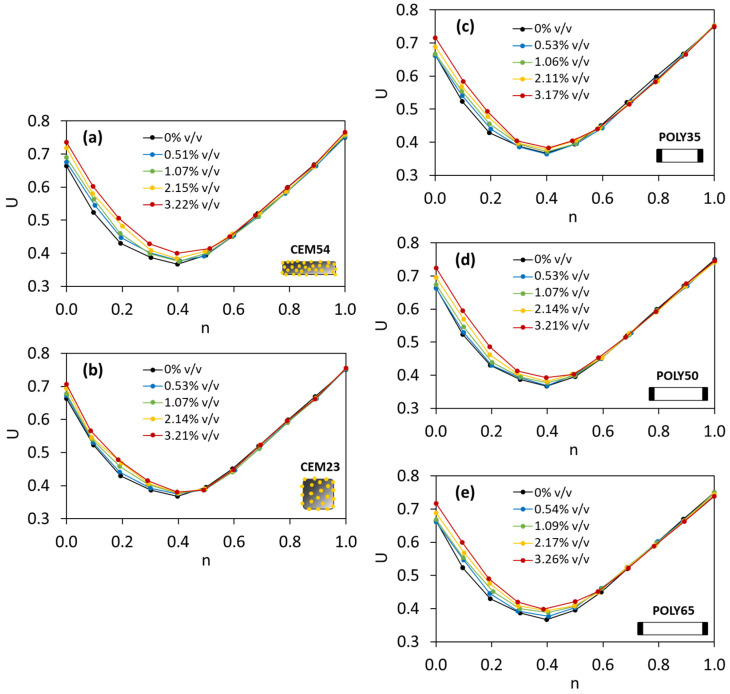
Voids ratios of TAM with (**a**) CEM54 capsules, (**b**) CEM23 capsules, (**c**) POLY35 capsules, (**d**) POLY50 capsules and (**e**) POLY65 capsules at different fine fractions based on the lab results.

Furthermore, the CEM54 capsules were added to the TAM to investigate the effect of long capsules toward the change in *U*, and the interaction diagram can be found in Figure 18a. It is evident that the addition of capsules into the TAM certainly increased the voids ratio of TAM. The higher the capsule dosage, the higher the voids ratio. Moreover, the interesting fact here was projected at *n* of 0.6. TAM with *n* < 0.6 showed that the gradual addition of capsule tended to increase the voids ratio. In contrast, when *n* ≥ 0.6, the addition of capsules did not affect the voids ratio as the *U* values were identical to those of *U* of TAM without capsules (Figure 18a, black line). These findings suggest that the greater the content of coarse aggregates in the mixture (*n* < 0.6), the more disturbance in the packing system due to capsule presence, whereas the greater the content of fine aggregates in the mixture (*n* ≥ 0.6), the less disturbance in the packing system. To confirm this finding, the other capsule types (CEM23, POLY35, POLY50 and POLY65) were also added to TAM, and the interaction diagrams can be found in Figure 18b–e. The use of CEM capsules resulted in the same behaviour as the use of POLY capsules.

Comparing the influence of CEM23 and CEM54 capsules in the interaction diagram, the difference between *U* values as a function of capsule dosage, specifically at *n* between 0 and 0.6, was much bigger with long capsules than with short ones (see Figure 18a,b). From Figure 18c–e, it is also clear that changing the POLY capsule length from 35 to 50 or 65 mm affected the *U* values when TAM was fixed with *n* in the same range. Consequently, the use of short capsules can be more beneficial than the use of long capsules in terms of reducing the disturbance of the packing.

**Figure 19:**
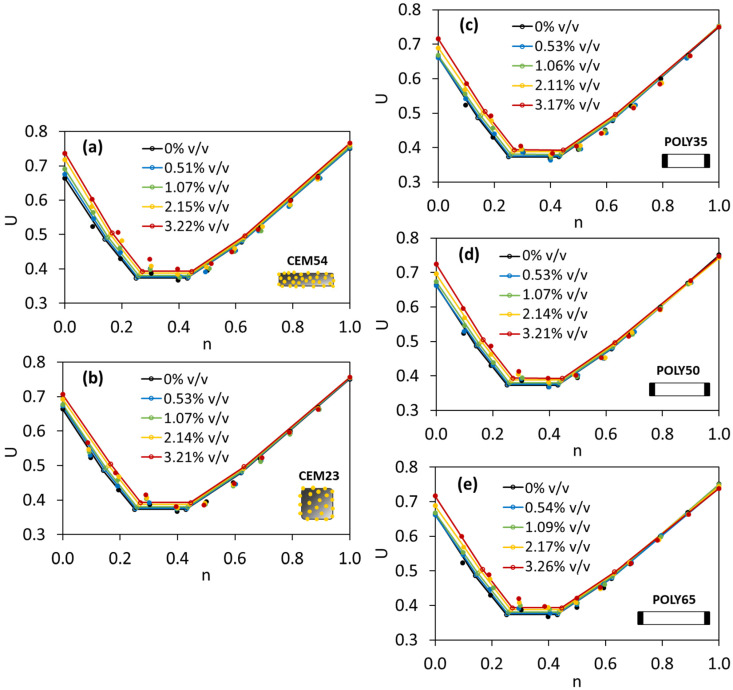
Voids ratios of TAM with (**a**) CEM54 capsules, (**b**) CEM23 capsules, (**c**) POLY35 capsules, (**d**) POLY50 capsules and (**e**) POLY65 capsules at different fine fractions based on the Dewar results as compared to the lab results (note: straight line with hollow dot represents Dewar result and solid dot represents experimental/lab result).

The particle packing model of Dewar was implemented for TAM with capsules, and the comparison of interaction diagrams between experimental and Dewar results can be seen in Figure 19. When the capsules were added, regardless of their type and dosage, the results showed a good compatibility between experimental and Dewar results. The lowest *U* was found at the fine fraction of 0.4 based on lab results (see Figure 18) and is comparable with Dewar’s result with the fine fraction of around 0.43 (see Figure 19). Regardless of capsule type and capsule dosage, the lowest *U* values remained the same as that for TAM without capsules, indicating the same optimal aggregate composition in all cases.

**Figure 20:**
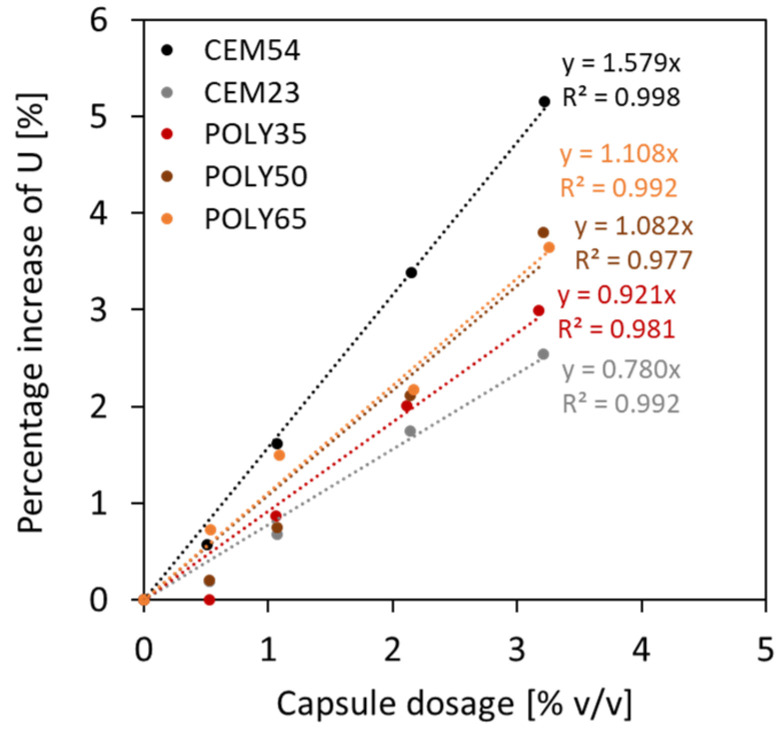
Relationship between capsule dosage and percentage increase in voids ratio observed in TAM.

**Figure 21:**
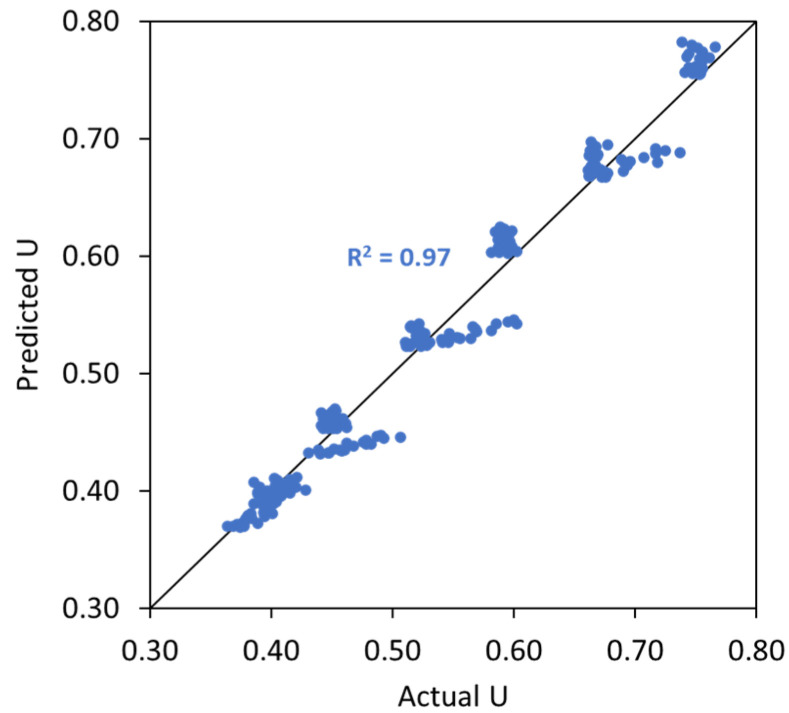
Comparison between the actual *U* values and the predicted *U* values of TAM as a function of capsules dosage and *L_caps_*/*D_caps_* ratio.

As a matter of fact, the particle packing model of Dewar does not automatically calculate the effect of capsules as the original model is normally intended for design of normal concrete. Although there is a good fit between experimental and Dewar results, the model should be upgraded for the introduction of the capsules. This can be achieved by formulating the voids ratio of TAM as a function of the capsule *L_caps_*/*D_caps_* ratio and capsule dosage. To realise this, the same modelling process from BAM was implemented in TAM. A relationship between the voids ratio for 0% *v*/*v* capsules and the voids ratio at a certain capsule dosage was constructed for each specific capsule type, and the percentage increment in *U* relative to capsule dosage could be established, as depicted in Figure 20. It was also confirmed that the effect of long capsules was more dominant than that of short capsules. The results from Figure 20 can be charted and normalised based on the *L_caps_*/*D_caps_* ratios. Finally, the following formula is proposed to predict the voids ratio of TAM for the addition of all types of capsules with specific aggregates used in this study:

(11) $$U_{TAM\_caps}=U_{TAM\_initial}\left((0.0004\frac{L_{caps}}{D_{caps}}+0.009)d_{caps}+1\right)$$

where $U_{TAM\_caps}$ is the voids ratio of TAM after capsules’ addition, $U_{TAM\_initial}$ is the initial voids ratio of TAM without any capsule, $d_{caps}$ is the dosage of capsules [% *v*/*v*], $L_{caps}$ is the length of the capsules [mm], and $D_{caps}$ is the outer diameter of the capsules [mm]. To validate the robustness of Equation (11), the ‘actual’ voids ratios from lab experimental tests (from Figure 18) were compared with the ‘predicted’ results based on the calculation of $U_{TAM\_caps}$ considering the capsules’ parameters and $U_{TAM\_initial}$ (i.e., the voids ratio of TAM with 0% capsules). Figure 21 depicts the closeness of the values between the actual *U* and predicted *U*. The *R*^2^ was further calculated to highlight the similarities between the *U* values and the bisector line (as a note, the bisector line represents the same value between actual *U* and predicted *U*). Results showed that *R*^2^ reached 0.97, which also confirms that the above equation can be used to predict the voids ratio of TAM as a function of the capsule dosage and *L_caps_*/*D_caps_* ratio with good accuracy. Furthermore, validation was conducted to show that the deviations are acceptable, as explained in Section 4.5.

The effect of capsules in the packing of TAM was found to be much lower than the packing of BAM. This occurs because, in the packing of BAM (gravel–gravel), the presence of capsules generated extra voids between capsules and coarse particles. In the case of TAM, these extra voids are also present, but since fine particles (sand) are added to the mixture, those extra voids from the capsule–gravel interaction are filled with sand if available in a sufficient amount. This notion explains that in TAM, the effects of capsules are negligible, especially in the aggregate mixtures with *n* above 0.6, while with *n* below 0.6, there is a slight effect of the capsules as the aforementioned extra voids are not fully filled with the sand. Based on these interaction tests, the interaction between capsules and coarse aggregates is more important than the interaction between capsules and (fine + coarse) aggregates. In addition, the packing disturbance can be potentially minimised by formulating the inert structure with a high content of fine particles over coarse particles, in combination with tubular macrocapsules.

### 4.4. Validation of the Regression Model for the Factor k vs. (D_agg_/D_caps_)^2^(L_caps_/D_agg_)

**Figure 22:**
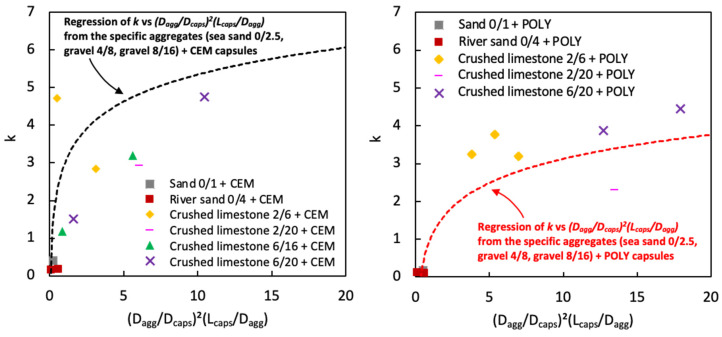
Comparison of the model for *k* vs. (*D_agg_*/*D_caps_*)^2^(*L_caps_*/*D_agg_*) with various aggregate types/fractions.

From Section 4.1, there was a clear relationship between the factor *k* vs. (*D_agg_*/*D_caps_*)^2^(*L_caps_*/*D_agg_*), as constructed via a logarithmic regression in Figure 10. However, the constraint of this relationship is the dependency on the specific aggregate types/fractions used (i.e., sea sand 0/2.5, gravel 4/8 and gravel 8/16) and capsule types (i.e., CEM and POLY). To validate the results, the relationship was tested with several combinations of other aggregate types (i.e., sand 0/1, river sand 0/4, crushed limestone 2/6, crushed limestone 2/20, crushed limestone 6/16, crushed limestone 6/20) in combination with CEM and POLY capsules, as illustrated in Figure 22. It is apparent that the previous relationship does not fit well with the other aggregate types/fractions. There are two reasons to explain this tendency: (1) the factor *k* can be sensitive to change with varying the aggregate type, aggregate fraction, capsule type and capsule size, and (2) a different capsule effect occurs with different aggregate types and fractions, leading to a fluctuation in the results between factor *k* vs. (*D_agg_*/*D_caps_*)^2^(*L_caps_*/*D_agg_*).

**Figure 23:**
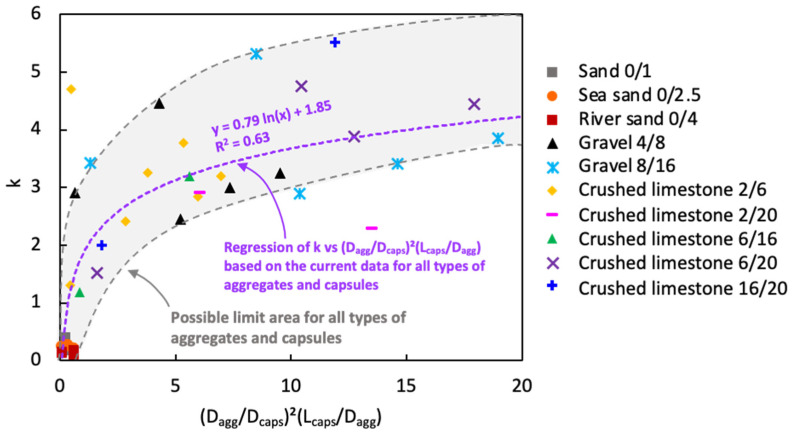
New relationship between factor *k* and (*D_agg_*/*D_caps_*)^2^(*L_caps_*/*D_agg_*) with various aggregate types/fractions.

To observe the (independent) variation aside from capsule types, Figure 23 was made by combining all data for different aggregate types and fractions. The best fitting curve in this case was a logarithmic regression with a moderate *R*^2^ value. Some data points were actually far from the regression, raising concerns regarding the reliability of the test results. It is interesting to observe that within the spread of data points, there are some points at the top and bottom whose envelopes could identify upper and lower limit curves, respectively. Potentially, this may be an indication of the limit area for the relation between the factor *k* vs. (*D_agg_*/*D_caps_*)^2^(*L_caps_*/*D_agg_*) for all types of aggregates and capsules. As a note, two data points outside the limit area were considered as outliers. Chu et al. [36] previously mentioned that the relation between the factor *k* vs. (*G*/*D_fibre_*)^2^(*L_fibre_*/*G*)^1.5^ was meant to set a limit on the aggregate size to avoid an excessive decrease in packing density when steel fibres are added. Since the effect of fibres is not the same as the effect of capsules, the limit area found in this study may potentially serve as a guideline to carefully select the aggregates (with *D_max_* < 20mm) for the inert structure of capsule-based concrete. Nevertheless, further research is certainly needed to enhance the model by using a wider variety of capsules and aggregates than used in this study.

### 4.5. Validation of the ‘U Model’ for Capsules

Following the analyses of capsules’ effect in different aggregate mixtures (from Section 4.2 and Section 4.3), the above formulas can be simplified as follow:

(12) $$U_{AM\_caps}=U_{AM\_no\;caps}\left((\alpha \frac{L_{caps}}{D_{caps}}+\beta )d_{caps}+1\right)$$

where $U_{AM\_caps}$ is the voids ratio of the aggregate mixture (AM) after capsules’ addition, $U_{AM\_nocaps}$ is the initial voids ratio of AM without any capsules (from experimental or Dewar results), $d_{caps}$ is the dosage of capsules [% *v*/*v*], $L_{caps}$ is the length of the capsules [mm], $D_{caps}$ is the outer diameter of the capsules [mm], and α and β are empirical constants depending on the number of combined aggregates (only two or three) with a maximum aggregate size ($D_{max}$) of 20 mm (currently restricted to 20 mm as no validation tests were conducted for $D_{max}$ > 20 mm) (see Table 6).

**Figure 24:**
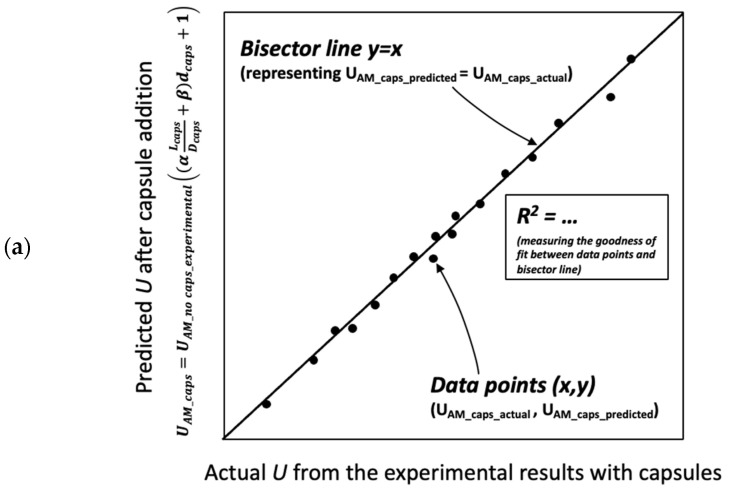

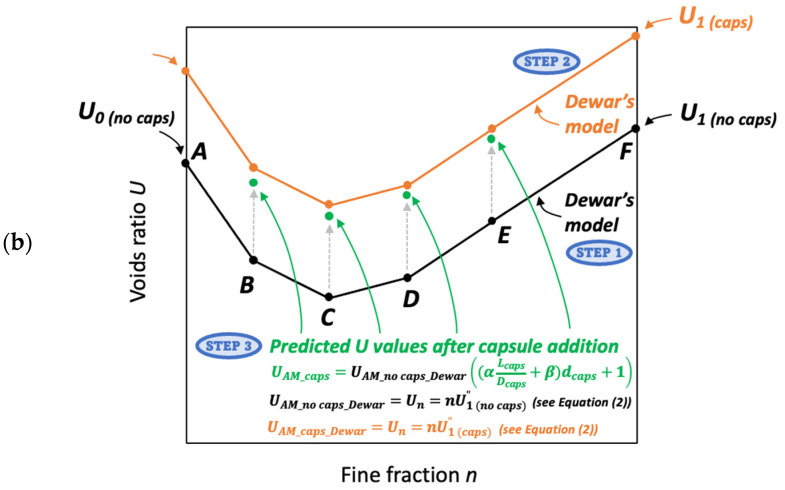
Validation scenarios to analyse the robustness of the ‘*U* model’: (**a**) actual *U* from the experimental results vs. predicted *U* from the model and (**b**) computed *U* from Dewar’s model vs. predicted *U* from the model.

The aforementioned formula, either BAM or TAM, has been proven to be robust for the aggregates specifically used in the study. There are two research questions that need to be addressed: (i) whether the ‘*U* model’ will be valid for the other materials (as defined in Section 4.4), and (ii) whether the ‘*U* model’ for capsules is compatible with the results from Dewar’s model. Consequently, two validation scenarios were developed, as shown in Figure 24, and the details are given below:

- The first scenario (Figure 24a) aims to validate the ‘*U* model’ based on the experimental approach. In this case, the actual (or measured) *U* values from the experimental results with capsules are compared with the predicted *U* values by computing the *U* values of aggregate mixtures without capsules (experiment-based) using the *U* values of aggregate mixtures with capsules via Equation (12). Finally, the statistical goodness-of-fit (*R*^2^) between the actual *U* and the predicted *U* is evaluated to justify the robustness of the ‘*U* model’.
- The second scenario (Figure 24b) aims to validate the ‘*U* model’ based on Dewar’s particle packing model. In Dewar’s model (step 1, black), the voids ratio diagram is constructed by calculating the points A–F based on *U*_0_ and *U*_1_ of single aggregates without capsules. Next, when the capsules are added, *U*_0_ and *U*_1_ of single aggregates are corrected by experimental tests (step 2, orange), resulting in an increase in *U* values at all points, as determined by application of Dewar’s model. It should be noted that the ratio of mean sizes (*r*) stays the same as in steps 1 and 2 because the capsules are not seen as aggregates; instead, they are considered as ‘barriers’ among aggregates that disturb the packing. In step 3, the *U* values of aggregate mixtures are computed via Equation (12) at the points B−E (green) and compared with the *U* values from Dewar’s model (from step 2, orange). In this case, the closeness of data points (B, C, D, E) is evaluated to justify the robustness of the ‘*U* model’.

To validate the ‘*U* model’ in different scenarios, several aggregate mixtures were defined with completely different aggregate–capsule formulations than the ones previously used in Section 4.2 and Section 4.3, as outlined in Table 7. The main objective was to verify the accuracy of the ‘*U* model’ with different types of aggregates and capsules.

#### 4.5.1. Validation of the ‘*U* Model’ Based on the Experimental Approach (the First Scenario)

The same experimental procedure for determining the *U* of aggregate mixtures without and with capsules was followed. The validation results are summarised in Table 8, showing the robustness of the ‘*U* model’ with good accuracy (*R*^2^ > 0.90). The results also prove that the ‘*U* model’ for capsules works well with other combinations of aggregates and capsules when considering the experimental *U* values. It should be noted that the empirical constants should be applied specifically for the designed mixtures. This means that if a mixture of two aggregate types/fractions is used, the empirical constants for BAM should be taken and they will not work for TAM (a mixture of three aggregate types/fractions), and vice versa. The limitation of BAM is that the aggregate mixture (with a continuous fraction) should be a combination of coarse–coarse or fine–coarse aggregates, while the combination of fine–fine aggregates is not intended. On the other hand, the limitation of TAM is that a mixture of three aggregates should be composed of fine–coarse–coarse or fine–fine–coarse aggregates (with a continuous fraction), which are typically used in practice for concrete mix design. If the fractions of the aggregates in the mixtures do overlap, additional study must be carried out for verification. Furthermore, the maximum aggregate size considered here was limited to 20 mm, while for bigger sizes, the model should be tested in the future. As this is the first study conducted in this area, it is recommended to undertaken a verification of the model when applying other aggregates/capsules.

#### 4.5.2. Validation of the ‘*U* Model’ Based on Dewar’s Modelling Approach (the Second Scenario)

**Figure 25:**
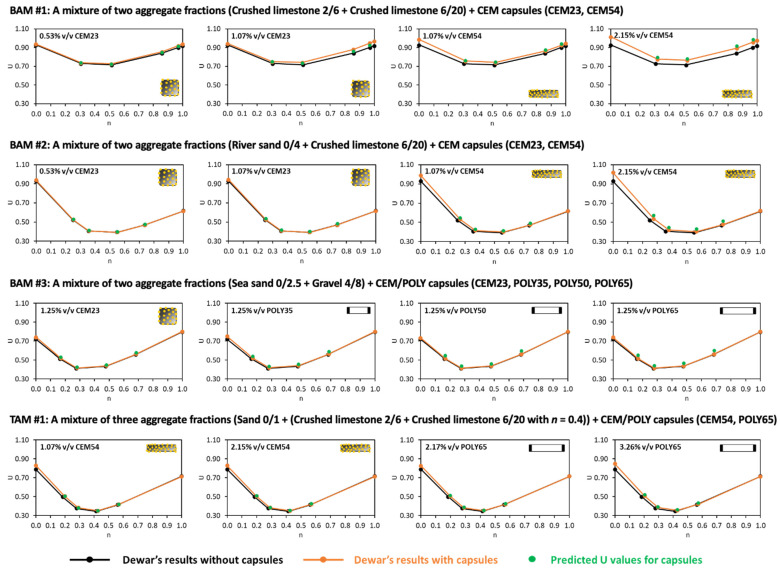
Interaction diagrams based on Dewar’s model and the ‘*U* model’ for capsules.

The ‘*U* model’ was validated with respect to Dewar’s model, as shown in Figure 25, using the combination of sixteen aggregate–capsule mixtures from Table 7. It is obvious that the voids ratio diagrams of aggregate mixtures with capsules (orange lines) slightly increased from the original voids ratio diagrams of aggregate mixtures without capsules (black lines) due to the disturbance created by the capsules. Overall, the predicted *U* values for aggregate–capsule mixtures at points B–E (green dots) matched very well with Dewar’s results with capsules (orange dots). This is evident from all combinations from Figure 25, regardless of capsule size, capsule dosage or aggregate mixture; thus, the robustness of the ‘*U* model’ is showcased. Although there were few offset points at certain combinations (e.g., BAM #2 + 2.15% *v*/*v* CEM54, BAM #3 + 1.25% *v*/*v* POLY65), the differences were rather minor. It was calculated that the largest difference and the mean difference between predicted *U* values and Dewar’s *U* values were approximately 7% and 3%, respectively, demonstrating a high prediction accuracy.

Based on these two validation scenarios, it was confirmed that the ‘*U* model’ for capsules from Equation (12) is relatively robust in the prediction of the change in the voids ratio of aggregate mixtures due to the capsules’ addition. As a note, the input of $U_{AM\_no\;caps}$ from Equation (12) can be taken from either the experimental results without capsules or Dewar’s results without capsules, as both of them showed good precision when the results were converted into the results with capsules.

## 5. Conclusions

This paper discusses the influence of tubular macrocapsules in the inert structure of self-healing concrete and attempts to adapt the particle packing model of Dewar for the introduction of macrocapsules. Several tubular macrocapsules were used with different shell materials and geometries. Various aggregates types and fractions were also adopted to understand the interaction between aggregates and capsules. The effect of macrocapsules was implemented in the particle packing model of Dewar to predict the voids ratio of aggregate mixtures, which can be useful for designing the inert structure of self-healing concrete. The main findings of this study are presented as follows:

(1) The introduction of macrocapsules did not alter the voids ratio of fine aggregates, but considerably increased the voids ratio of coarse aggregates. A higher capsule dosage led to a higher voids ratio of coarse particles due to the secondary loosening and wall effects induced by the capsules.
(2) The use of short capsules may be beneficial in terms of packing as they can blend well with aggregates in comparison with long capsules. Meanwhile, the use of crushed limestones exerted a higher impact on the packing with capsules than gravels due to their shape and surface roughness.
(3) A slight mismatch between experimental and Dewar results from the aggregate mixtures (without capsules) can sometimes be found, which can be corrected with an adjustment factor to increase the accuracy of Dewar’s model.
(4) The greater the content of coarse aggregates present in the inert structure, the larger the disturbance of the packing system due to the capsules. Therefore, the capsules’ effect can be minimised by using a high content of fine aggregates.
(5) The voids ratio of aggregates in the presence of tubular capsules was successfully predicted by considering the capsule parameters (i.e., capsule dosage, capsule length, capsule diameter and empirical constants). The limitations of the proposed and currently validated ‘*U* model’ for aggregate mixtures with capsules are that (i) the aggregate mixtures should be composed of two or three aggregate types/fractions with a continuous grading and (ii) the maximum aggregate size is 20 mm. Validation/extension of the model for other materials (combinations) needs further research.

## Figures and Tables

**Table 1:** Parameters for the change points in Dewar’s model [23].

Change Point	Parameters		
	*m*	*k_int_*	*k_p_*
A (*n* = 0)	0	-	-
B	0.3	0.12	0.60
C	0.75	0.06	0.65
D	3	0.015	0.8
E	7.5	0	0.9
F (*n* = 1)	∞	-	-

**Table 2:** Detailed geometry of the macrocapsules (based on the average values).

Capsule Name	Length, *L_caps_* [mm]	Outer Diameter, *D_caps_* [mm]	Inner Diameter [mm]	*L_caps_*/*D_caps_* Ratio	Mass [g]	Volume [mm^3^]
CEM23	22.73	14.97	12.00	1.52	6.05	4000
CEM54	54.19	9.06	6.00	5.98	5.98	3490
POLY35	35.61	6.65	6.01	5.35	0.81	1240
POLY50	50.10	6.65	6.01	7.53	1.07	1740
POLY65	65.03	6.65	6.01	9.78	1.33	2260

**Table 3:** Properties of aggregates.

Aggregate	Oven-Dry Particle Density, *ρ_rd_* [kg/m^3^]	Loose Bulk Density, *ρ_b_* [kg/m^3^]	Voids Ratio, *U* [-]	Mean Size [mm]
Sea sand 0/2.5	2670	1520	0.751	0.40
River sand 0/4	2690	1630	0.656	0.90
Red sand 0/4	2640	1660	0.592	0.80
Gravel 4/8	2600	1550	0.672	6.48
Gravel 8/16	2600	1490	0.742	12.88
Crushed limestone 2/6	2630	1350	0.952	4.30
Crushed limestone 6/16	2640	1410	0.877	8.47
Crushed limestone 16/20	2660	1400	0.905	18.00

**Table 4:** Number of capsules used for LBD tests.

CEM23		CEM54		POLY35		POLY50		POLY65	
No. of Caps. [pcs]	Caps. Dosage [% *v*/*v*]	No. of Caps. [pcs]	Caps. Dosage [% *v*/*v*]	No. of Caps. [pcs]	Caps. Dosage [% *v*/*v*]	No. of Caps. [pcs]	Caps. Dosage [% *v*/*v*]	No. of Caps. [pcs]	Caps. Dosage [% *v*/*v*]
0	0	0	0	0	0	0	0	0	0
10	0.54	11	0.51	9	0.15	7	0.16	5	0.15
20	1.07	23	1.07	40	0.66	28	0.65	22	0.66
40	2.14	46	2.15	76	1.26	54	1.256	42	1.27
60	3.21	69	3.22	152	2.51	108	2.51	83	2.50
				212	3.50	151	3.51	116	3.50

**Table 5:** Assessment of the change in voids ratio of coarse aggregates due to the presence of CEM capsules (note: the increment of *k* = (*k_CEM54_* − *k_CEM23_*)/*k_CEM23_* × 100%).

Aggregate	Factor *k* [%/(% *v*/*v*)]		Increment of *k* [%]
	CEM23	CEM54	
Gravel 4/8	2.90	4.47	54
Gravel 8/16	3.42	5.32	56
Crushed limestone 2/6	1.31	2.41	84
Crushed limestone 6/16	1.18	3.20	171
Crushed limestone 16/20	2.00	5.52	176

**Table 6:** Empirical constants for $U_{AM\_caps}$.

AM Type	α	β
A mixture of two aggregate types/fractions (= BAM) (e.g., gravel 4/8 + gravel 8/16)	0.004	0.02
A mixture of three aggregate types/fractions (= TAM) (e.g., sand 0/2.5 + gravel 4/8 + gravel 8/16)	0.0004	0.009

**Table 7:** Composition of aggregate mixtures for validation purposes.

Aggregate Mixture (AM)		Fine Fraction (*n*)	Capsule Type and Dosage (*d_caps_*)
BAM #1	Crushed limestone 2/6 + Crushed limestone 6/20	0, 0.2, 0.4, 0.6, 0.8, 1.0	CEM23: 0, 0.53, 1.07% *v*/*v* CEM54: 0, 1.07, 2.15% *v*/*v*
BAM #2	River sand 0/4 + Crushed limestone 6/20	0, 0.2, 0.4, 0.6, 0.8, 1.0	CEM23: 0, 0.53, 1.07% *v*/*v* CEM54: 0, 1.07, 2.15% *v*/*v*
BAM #3	Sea sand 0/2.5 + Gravel 4/8	0, 0.2, 0.4, 0.6, 0.8, 1.0	CEM23: 0, 1.25% *v*/*v* CEM54: 0, 1.25% *v*/*v* POLY35: 0, 1.25% *v*/*v* POLY50: 0, 1.25% *v*/*v* POLY65: 0, 1.25% *v*/*v*
TAM #1	Sand 0/1 + (Crushed limestone 2/6 + Crushed limestone 6/20 with *n* = 0.4)	0, 0.2, 0.4, 0.6, 0.8, 1.0	CEM54: 0, 0.53, 1.07% *v*/*v* POLY65: 0, 1.07, 2.15% *v*/*v*

**Table 8:** Validation results.

Aggregate Mixture (AM)			
BAM #1:	Crushed limestone 2/6 + Crushed limestone 6/20 + All CEM capsules 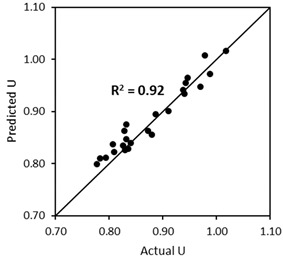	BAM #3:	Sea sand 0/2.5 + Gravel 4/8 + All capsules types (CEM and POLY) 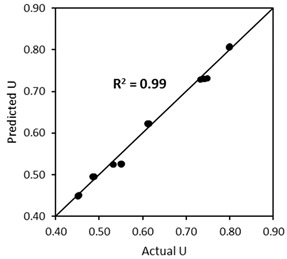
BAM #2:	River sand 0/4 + Crushed limestone 6/20 + All CEM capsules 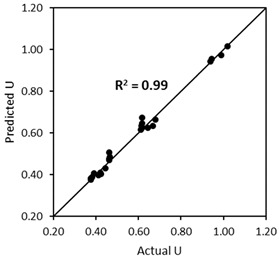	TAM #1:	Sand 0/1 + (Crushed limestone 2/6 + Crushed limestone 6/20 with *n* = 0.4) + two specific types of capsules (CEM54 and POLY65) 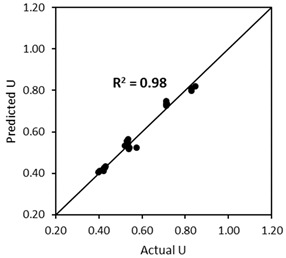

## Data Availability

Data related to the publication will be available on the SMARTINCS Zenodo platform.
